# Transgenerational plasticity as an important mechanism affecting response of clonal species to changing climate

**DOI:** 10.1002/ece3.3105

**Published:** 2017-06-07

**Authors:** Zuzana Münzbergová, Věroslava Hadincová

**Affiliations:** ^1^ Department of Botany Faculty of Science Charles University Prague Czech Republic; ^2^ Institute of Botany Academy of Sciences of the Czech Republic Průhonice Czech Republic

**Keywords:** climatic extremes, common garden experiment, epigenetic memory, *Festuca rubra*, genome methylation, local adaptation, reciprocal transplant experiment

## Abstract

In spite of the increasing number of studies on the importance of transgenerational plasticity for species response to novel environments, its effects on species ability to respond to climate change are still largely unexplored. We study the importance of transgenerational plasticity for response of a clonal species *Festuca rubra*. Individuals from four natural populations representing two levels of temperature and two levels of precipitation were cultivated in four growth chambers that simulate the temperature and precipitation of origin of the populations (maternal phase). Each population was represented in each growth chamber. After 6 months, single young ramets of these plants were reshuffled among the growth chambers and let to grow for additional 2 months (offspring phase). The results show that transgenerational effects (i.e., maternal phase conditions) significantly modify species response to novel climates, and the direction and intensity of the response depend on the climate of origin of the plants. For traits related to recourse acquisition, the conditions of maternal phase, either alone or in interaction mainly with climate of origin, had stronger effect than the conditions of cultivation. Overall, the maternal climate interacted more intensively with the climate of origin than with the offspring climate. The direction of the effect of the maternal climate was of different directions and intensities depending on plant origin and trait studied. The data demonstrated strong significant effects of conditions during maternal phase on species response to novel climates. These transgenerational affects were, however, not adaptive. Still, transgenerational plasticity may be an important driver of species response to novel conditions across clonal generations. These effects thus need to be carefully considered in future studies exploring species response to novel climates. This will also have strong effects on species performance under increasingly variable climates expected to occur with the climate change.

## 1 INTRODUCTION

Global climate change represents a strong pressure on natural populations (Guillaume, Monro, & Marshall, 2016). It is generally accepted that species response to such a change strongly depends on species ability to migrate, undergo adaptive genetic change, or exhibit phenotypic plasticity (Hoffmann & Sgro, 2011). Recently, it has been suggested that also transgenerational plasticity may be an important mechanism allowing the species to deal with changing climate. The studies explicitly linking transgenerational plasticity with species response to changing climate are dealing mainly with marine organisms (e.g., Donelson, Munday, McCormick, & Pitcher, 2012; Guillaume et al., 2016; Munday, Warner, Monro, Pandolfi, & Marshall, 2013; Sunday et al., 2014) and insects (Sgro, Terblanche, & Hoffmann, 2016), with few studies also dealing with plants (e.g., Germain & Gilbert, 2014; Herman & Sultan, 2016; Walter, Harter, Beierkuhnlein, & Jentsch, 2016). As plants are sessile organisms in contrast to the other groups usually studied, plants have lower chances to migrate to suitable environments and transgenerational plasticity may thus be more important in their populations. This is even more true in long‐lived plant species and plant species with prevailing vegetative reproduction.

The concept of transgenerational plasticity builds upon the recognition that parent individuals may alter specific developmental traits in their progeny in response to particular environmental stresses and that these alterations may enhance offspring growth and success under those same stresses (Allen, Buckley, & Marshall, 2008; Herman & Sultan, 2011). The transgenerational effects may be mediated by direct response to reduced provisioning by resource‐deprived parents (Roach & Wulff, 1987). Alternatively, they may be mediated by other, more stable mechanisms leading to heritable epigenetic changes within the organisms (e.g., Bossdorf, Richards, & Pigliucci, 2008; Cortijo et al., 2014; Herman & Sultan, 2016; Kappeler & Meaney, 2010). While the transgenerational effects seem to be potentially important drivers of species response to changing climates, the effects may not always be positive and their direction and magnitude may depend on the specific environmental stresses and their variation taking place (Guillaume et al., 2016).

The existing studies dealing with transgenerational plasticity are mainly concerned with stresses such as nutrient deficiency, high salinity, herbivory, and viral infections (reviewed by Herman & Sultan, 2011). A range of recent studies, however, also looked at the importance of transgenerational plasticity for species response to variable climate, represented either by pulses of drought or high temperatures or their between season variation (reviewed by Herman & Sultan, 2011), later (e.g., Herman & Sultan, 2016; Herman, Sultan, Horgan‐Kobelski, & Riggs, 2012; Walter et al., 2016). These studies (e.g., Germain & Gilbert, 2014; Herman & Sultan, 2011, 2016; Herman et al., 2012; Walter et al., 2016) usually explore the effects of a single well‐defined climatic factor. The aim of these studies is not to understand the effects of climate change, but rather the effects of these specific treatments and single‐factor manipulations are thus useful approaches to answer their questions of interest. Climate change is, however, not a unidirectional change in one climatic factor alone, but is likely to bring about novel combinations of precipitation, temperatures, and their fluctuations (IPCC, 2014). Understanding the effects of the different climate combinations is thus important for proper calibration of models predicting species response to future climatic changes (Elith & Leathwick, 2009; Gotelli & Stanton‐Geddes, 2015; Meineri, Deville, Gremillet, Gauthier‐Clerc, & Bechet, 2015; Moran, Hartig, & Bell, 2016; Parmesan & Hanley, 2015). Nevertheless, studies exploring the importance of transgenerational plasticity in response to multiple climatic factors simultaneously are still missing.

Most of the research on transgenerational plasticity in relationship to climate in plants has been performed on annual species (e.g., Germain & Gilbert, 2014; Herman & Sultan, 2016; Leverett, Auge, Bali, & Donohue, 2016; Penfield & Springthorpe, 2012; Suter & Widmer, 2013a, 2013b; Whittle, Otto, Johnston, & Krochko, 2009). Transgenerational plasticity may, however, be even more important in perennial species with long life cycles. In such species, genetic adaptation by natural selection could be too slow to keep pace with rapid climate change, increasing the importance of other mechanisms such as plasticity or transgenerational effects (Walter et al., 2016). However, only very few studies dealing with the importance of transgenerational plasticity for plant response to different climatic factors were in fact performed on perennials (e.g., Bernareggi, Carbognani, Petraglia, & Mondoni, 2015; Mondoni et al., 2014; Walter et al., 2016). In addition, while the majority of the existing studies compared transgenerational plasticity between maternal plants and their offspring originating from seeds, it has been recently suggested that similar patterns can be observed between maternal plants and their clonal offspring (Dalrymple, Buswell, & Moles, 2015; Gonzalez et al., 2016; Latzel & Klimesova, 2010; Verhoeven & Preite, 2014). Verhoeven and Preite (2014) suggested that epigenetic variation may be more important in populations of clonally reproducing species as it represents an important mechanism allowing them to adapt to environmental variation with somatic mutations being the only other option (see Barrett, 2015). Furthermore, clonal reproduction circumvents meiosis, which is associated with resetting epigenetic memory among populations (Verhoeven & Preite, 2014). Our knowledge on the importance of transgenerational effects for adaptation to climate in populations of clonal plants is very sparse even though clonal plants represent dominants in many terrestrial systems (Klimeš, Klimešová, Hendriks, & Van Groenendael, 1997), and their ability to adapt to changing climate will likely affect not only their own populations, but functioning of the whole ecosystems.

Most of the experiments dealing with transgenerational plasticity are concerned with plants of single origin often without providing any information on the population of origin (e.g., Germain & Gilbert, 2014; Latzel et al., 2013; Walter et al., 2016). Alternatively, studies use several different lineages of the same species and often show very different patterns of transgenerational plasticity among these (Bernareggi et al., 2015; Herman & Sultan, 2016; Lampei, Metz, & Tielborger, 2017; Mondoni et al., 2014; Penfield & Springthorpe, 2012; Suter & Widmer, 2013a, 2013b; Vu, Chang, Moriuchi, & Friesen, 2015). Of these, only Mondoni et al. (2014), Bernareggi et al. (2015), and Lampei et al. (2017), however, explicitly evaluated the condition of origin of the population compared to the cultivating conditions. Except for Lampei et al. (2017), they only had two origins and one cultivating environment. Further studies exploring the effect of conditions of origin on the magnitude and direction of transgenerational plasticity are thus needed to uncover to what extent these effects depend on plant origin. Such knowledge is crucial for our ability to predict response of widespread species to novel conditions.

The aim of this study was to assess the importance of transgenerational plasticity among asexual generations of a clonal grass, *Festuca rubra*, in response to variable climate. We did so using four populations originating from contrasting climatic conditions in western Norway representing the four climatic extremes of a unique natural grassland “climate grid” spanning ~4°C in temperature and ~2,100 mm in precipitation established in western Norway (the SeedClim grid, see Klanderud, Vandvik & Goldberg 2015; Meineri, Skarpaas & Vandvik 2012; Meineri, Spindelbock & Vandvik 2013; Meineri, Skarpaas, Spindelbock, Bargmann, & Vandvik, 2014). The climatic prediction for Norway suggests increases in both precipitation (by about 18%) and temperature (by about 1.5°C to 2.2°C) over the next century (Hanssen‐Bauer et al. 2005). Our experimental sites thus go beyond the expected climate change and provide the first test of the potential importance of transgenerational plasticity in response to temperature and precipitation in a clonal plant. In case of proving significant effects of these climatic variables, future studies should attempt to identify whether transgenerational effects also play a role in case of subtler climatic changes.

To study plant performance, we used a range of plant characteristics describing plant growth and resource acquisition. In a previous study in the system, we have shown that while the growth‐related traits are very plastic, the traits related to resource acquisition rather show strong differentiation between populations (Münzbergová et al. 2017). This study also suggested that number of ramets may be the best measure of plant fitness as the species is very long‐lived and clonal and number of ramets thus represents as good measure of production of new vegetative offspring (Münzbergová et al. 2017).

In the current study, we asked the following questions: (1) What is the effect of temperature and moisture of maternal generation on offspring response to actual temperature and moisture? (2) How does this response depend on temperature and moisture in natural conditions from which the plants originated? (3) Is transgenerational plasticity adaptive, that is, do the plants perform better in case of being exposed to same the maternal and offspring environments?

We hypothesize that temperature as well as moisture in the maternal generation will strongly impact plant performance. The variance explained by the maternal environment will be lower than the variance explained by cultivating conditions in highly plastic traits such as plant size. In contrast, in traits previously showing low plasticity (i.e., traits related to resource acquisition, Münzbergová et al. 2017), we expect that maternal environment will play much larger role. In addition, we expect that the effects of maternal environment will depend on population origin with plants from more extreme environments (i.e., colder and drier) showing weaker response to maternal environments (as these populations are more plastic, Münzbergová et al. 2017). Finally, we hypothesize that transgenerational plasticity will be adaptive, that is, plants will perform better in the given environment if they already experienced that environment in the maternal generation.

## 2 METHODS

### 2.1 Study system

We used *F. rubra* L. as a model species in our study. *Festuca rubra* L. is a common perennial grass species of temperate grasslands in Europe. In the experiment, we use *F. rubra* ssp. *rubra,* a widespread hexaploid type from the *F. rubra* complex. It reproduces by seeds as well as vegetatively, producing both intravaginal and extravaginal tillers on rhizomes. *Festuca rubra* possesses considerable genetic variability and plasticity (Herben, Krahulec, Hadincova, & Pechackova, 2001; Skalova et al., 1997). *Festuca rubra* is a long‐lived species. It has been estimated to live for several hundred years (Harberd, 1961; de Witte & Stocklin, 2010).

The experimental plants were collected from four most extreme localities along a natural climatic grid established in western Norway (the SeedClim Grid, see Klanderud et al. 2015). Specifically, we sampled four grassland localities representing two levels of summer temperature (means of the four warmest months for individual locality types; ca. 6.5°C [alpine, ALP] and 10.5°C [boreal localities, BOR]) and two levels of mean annual precipitation [ca. 600 and 2,700 mm]. Each climate combination is thus represented by a single population. The target communities are grazed intermediate‐rich meadows (Potentillo‐Festucetum ovinae; G8 sensu (Fremstad, 1997) occurring on southwest‐facing, shallow slopes (5–20°) with relatively rich bedrock in terms of nutrient availability. Sites were selected specifically to ensure that grazing regime and grazing history, bedrock, slope, aspect and vegetation types are as similar as possible (Meineri et al., 2014).

### 2.2 Experimental setup

For the study, we used six different clones collected at each natural locality at least 1 m apart. Šurinová et al. (unpubl.) confirmed that these clones were genetically differentiated from each other, thus representing true independent genotypes. We use the term genotype throughout the subsequent text. These genotypes were cultivated for 5 months in the experimental garden of the Institute of Botany, Czech Academy of Sciences, Průhonice, Czech Republic (49°59′38.972″N, 14°33′57.637″E; means of the four warmest months 16.5°C and regular watering during the vegetation season) prior to the experiment. Further, they were grown for two more months in a greenhouse heated to ensure that the temperature never drops below 10°C (for more details, see Munzbergova et al. 2017).

Four ramets (single‐plant tillers able of independent existence) of each of the six genotypes from each population have been individually planted into four growth chambers. The plants were grown in the growth chambers for a purpose of another experiment from mid‐March to end of August 2015 (Münzbergová et al. 2017). The description of the setup thus corresponds to the previous description provided in Münzbergová et al. 2017). We used climatic chambers (Vötch 1014) simulating four different scenarios for the spring to summer climate in the field (second half of April–second half of June). The four scenarios were derived from climate data for the four localities within the technical limits of the climatic chambers and avoiding night frosts (minimum temperature during cultivation being 3°C). The temperature in the growth chamber differed between the cold and warm treatments and changed over the growing season following the course of temperature at the natural localities (for details, see Table 1 in Münzbergová et al. 2017). To set the correct moisture level in the growth chambers, we used TMS5 data loggers to continuously measure soil moisture in the pots (TOMST Co., Hemrová, Knappová, & Münzbergová, 2016) and identified the correct level of watering to achieve soil moisture comparable to that at the localities. As a result of this calibration, the dry regime plants were watered with about 20 ml of tap water per plant applied to the trays if the soil moisture was lower than 15%. In the wet regime, plants were cultivated under full soil saturation with about 1.5 cm of water in the bottom of the tray. Soil moisture was monitored continuously during the whole experiment, and watering was modified to ensure constant moisture throughout the experiment. Three data loggers were placed in each growth chamber. Each data logger was placed in a pot with a growing *Festuca* plant, which was intermixed among the experimental plants and was of the same size as the experimental plants, but was not a part of the experiment. For all the regimes, the same day length and radiation were used, that is, 16 hr of full light (6 a.m.–10 p.m.) and 4 hr of full dark with a gradual change of light availability in the transition between the light and dark periods over 2 hr. Over the full light period, the radiation was 360 μmol m^−2^ s^−1^, red radiation (R, λ = 660 nm) of 26 μmol m^−2^ s^−1^, and far‐red radiation (FR, λ = 730 nm) of 15 μmol m^−2^ s^−1^, R/FR = 1.73 (the radiation measured using a SPh 2020 photometer from Optické dílny, Turnov, Czech Republic).

**Table 1:** Effect of temperature (Temp, T) and moisture (Mois, M) of origin (O), of the maternal phase (1) and of the offspring phase (2) of the experiment on all the measured species characteristics in the offspring phase (C2). Significant values (*p* ≤ .05) are in bold. Triple interactions of the variables are given in Table S2. Results marked with * are significant also after correcting for multiple testing. TO and MO thus represent the effects of origin, T1 and M1 represent the effects of C1, and T2 and M2 represent the effects of C2

	Plant height		Ramet no.		Below: aboveg.		Aboveg. biom.		Prop extrav. ramets	
	*F*	*p*	*F*	*p*	*F*	*p*	*F*	p	Chi	*p*
TempO	0.74	.390	0.15	.699	2.25	.134	0.26	0.607	0.11	.745
Temp1	**4.85**	.028*	**22.00**	<.001*	**40.69**	<.001*	**24.85**	<0.001*	**15.66**	<.001*
Temp2	**828.2**	.001*	**6.58**	.010*	**841.32**	<.001*	**163.1**	<0.001*	**26.47**	<.001*
MoisO	0.57	.449	0.55	.459	<0.01	.955	2.86	0.091	**10.73**	.001*
Mois1	0.08	.777	**4.30**	**.038**	1.21	.271	1.78	0.182	**34.09**	<.001*
Mois2	**66.08**	<.001*	**27.01**	<.001*	**47.03**	<.001*	**194.4**	<0.001*	**18.23**	<.001*
TO × T1	2.00	.158	**16.14**	<.001*	1.52	.218	1.42	0.234	**14.28**	<.001*
TO × T2	0.60	.440	1.13	.287	**3.90**	**.048**	0.07	0.790	0.73	.393
TO × MO	**10.42**	.001*	2.21	.137	**8.60**	.003*	0.79	0.373	2.07	.151
TO × M1	0.26	.609	0.86	.354	1.85	.173	0.37	0.542	<0.01	.962
TO × M2	**4.38**	**.036**	0.28	.595	0.34	.560	**7.99**	0.005*	0.40	.528
T1 × T2	0.55	.458	0.22	.641	3.22	.073	0.02	0.888	0.06	.812
T1 × MO	0.16	.693	1.26	.263	0.02	.889	<0.01	0.992	0.78	.377
T1 × M1	**4.93**	**.026**	0.01	.918	0.42	.515	**4.06**	**0.044**	0.17	.682
T1 × M2	0.11	.745	0.06	.800	0.56	.454	2.00	0.158	0.20	.651
MO × M1	0.35	.555	0.70	.404	0.01	.915	**5.43**	**0.020**	**5.44**	**.020**
MO × M2	**43.05**	<.001*	**4.51**	**.034**	0.80	.373	**33.44**	<0.001*	**10.01**	.002*
M1 × M2	0.21	.644	**3.59**	**.058**	0.04	.847	0.76	0.382	0.24	.622
MO × T2	**10.58**	.001*	**29.47**	<.001*	1.38	.239	**35.21**	<0.001*	2.42	.120
T2 × M1	0.67	.412	0.02	.900	1.79	.181	0.06	0.800	0.04	.843
T2 × M2	**220.5**	<.001*	0.48	.488	**5.37**	**.020**	**33.27**	<0.001*	**3.75**	**0.053**

The plants grew in 5 × 5 × 8.5 cm pots filled with a mixture of common garden soil and sand in 2:1 ratio. This cultivation represents maternal generation, and we refer to cultivation in this period as C1. After terminating the C1 experiment at the end of August 2015, 12 young ramets have been separated from each individual pot. The single young ramets were individually planted to pots (5 × 5 × 8.5 cm pots filled with a mixture of common garden soil and sand in 2:1 ratio). To account for the variation in biomass between the selected ramets, we recorded height of each planted ramet and used this height as a covariate in the subsequent tests. The roots of the ramets as well as the aboveground part of the ramets were then shortened to ensure that all the ramets have similar belowground as well as aboveground systems of 3 cm in length. Three pots containing ramets of each genotype and each C1 growth chamber were placed into each of the new four growth chambers, leading to 288 pots in each new growth chamber in total (3 pots per genotype × 6 genotypes × 4 original populations × 4 C1 growth chambers). The plants were let to grow in the growth chambers for 63 days. The conditions in the growth chamber corresponded to the conditions during the peak vegetation season, that is, the last temperature setting in the C1 generation, and were retained over the whole period. This second phase of cultivation represents the offspring generation and is further referred to as C2. At the end of the experiment, we recorded number of all ramets and measured length of the longest ramet (hereafter referred to as plant height). We also distinguished between intravaginal and extravaginal ramets and estimated the proportion of extravaginal ramets. Intravaginal ramets are those that develop within the subtending leaf sheath, resulting into minimal interramet distance. In contrast, the development of extravaginal ramet proceeds laterally through the subtending leaf sheath contributing to a greater interramet distance within a clone (Briske & Derner, 1998). Afterward, the plants were removed from the pots, and the belowground parts were carefully washed and sorted into roots and rhizomes. All the belowground as well as aboveground biomass was dried to a constant weight and weighted. The number of extravaginal ramets divided by the total number of ramets was calculated to give proportional data per plant. Proportion of extravaginal ramets and rhizome weight are two key traits describing foraging ability of the plant. Plants with more rhizomes and thus more extravaginal ramets extend the area from which they can acquire resources. This strategy is likely to improve the growth of the species in environments with limiting nutrients and/or water. Plant foraging can also be described by species investment to belowground biomass and especially by the ratio between belowground and aboveground biomass. The other plant characteristics, that is, aboveground biomass, plant height, and number of ramets, are primarily describing performance of the plant. We assume that number of ramets is the best proxy of plant fitness in this clonal species, as it provides information on number of new clonal offspring.

### 2.3 Data analyses

We tested the effects of temperature and precipitation of the original locality (further referred to as origin), target conditions in the maternal generation (C1), target conditions in the offspring generation (C2), and all their interactions on performance of the plants in C2. We used initial plant size (i.e., size after C1) as a covariate in preliminary tests. As it did not have any effects on plant performance due to its very small variation, it was discarded from the final tests. In all cases, the conditions of origin, C1 and C2, were coded separately by their temperature, precipitation, and their interaction. For visualization of the results, we, however, merged the effects of origin, C1 and C2, without distinguishing the species factors. All the tests were conducted using linear or generalized linear mixed‐effect models with plant genotype as a random factor using the package LME4 in R 3.2.1 (R Development Core Team, 2011). Specifically, the linear mixed‐effect model was used for all dependent variables except for proportion of extravaginal ramets and rhizome biomass. We used generalized linear mixed‐effect model assuming binomial and Gamma distribution for proportion of extravaginal ramets and rhizome biomass, respectively.

To assess whether C1 conditions increased performance of plants exposed to the same C2 conditions, we added codes coding whether C1 and C2 temperature and moisture were identical or not. We tested the effect of these codes alone and in interaction with original temperature and moisture on all the traits. We used the same types of tests as described above for these analyses. In case this test will show significant effect and plants experiencing the same conditions in both maternal (C1 conditions) and offspring generation (C2 conditions), we will conclude that transgenerational plasticity is adaptive.

To visualize the results, we calculated mean value of each plant characteristic across all individuals grown within each C2 growth chamber and subtracted the value from values of all individuals grown in that C2 growth chamber. In that way, we obtained deviations of each individual from mean performance of individuals in that C2 growth chamber. Thanks to this, we could easily compare plant performance across different origins and C1 cultivating conditions, without the necessity to visualize the effects of C2 conditions. While the effects of C2 are indeed also interesting and represent plastic responses of the species, they have already been studied in our previous study (Münzbergová et al. 2017) and their demonstration is not the aim of this study. For each plant origin and cultivating conditions in C1, we assessed whether the value of each plant characteristic significantly deviated from mean expected for the given C2 growth chamber, by testing whether the deviations significantly differed from zero using a *t* test. While these *t* tests have a high chance of type I, they are only meant to help in interpreting the figures. The proper tests of the data are provided in Table 1. The original data, showing also differences between the C2 growth chambers, are shown in Supporting information file.

Some of the dependent variables were closely correlated with each other (Table S1). From pairs of closely correlated variables (*r* > .7), we thus retained only one. Thanks to this, we excluded rhizome weight and belowground biomass from further analyses. All the above described tests were thus conducted for the following dependent variables: plant height, number of ramets, proportion of extravaginal ramets, total aboveground biomass, ratio between belowground and aboveground biomass.

To explore differences in the importance of the transgenerational plasticity in more detail, we tested the effects of C1 and C2 conditions in each population separately. We used these analyses to visualize the proportion of variance explained by C1 and C2 in each population.

In this study, we performed each test independently for five different traits measured on the same experimental plants. Theoretically, we should apply the Bonferroni correction and reduce the conventional *p* level from 0.05 to 0.01 (Dunn 1961). We decided to use a modification of this approach, the sequential Bonferroni correction (Holm–Bonferroni correction, Rice 1989) as it is considered as less conservative. Still any such correction is considered as too conservative by some authors (e.g., Garcia 2004; Gotelli & Ellison 2004; Moran 2003) and many studies have not applied any correction, for this reason (e.g., Bowman et al. 2008; Münzbergová 2007; Scheepens & Stocklin 2013). Here, we report and illustrate results both with and without this correction as we did also in our previous study on the system (Münzbergová et al. 2017)**.**

### 2.4 Methodological considerations

The setup of the experiment follows upon our previous study Münzbergová et al. (2017)**.** These methodological considerations reflect what has been previously written in Münzbergová et al. (2017). It may be argued that our experiment is pseudoreplicated as the growth chambers may theoretically differ in a range of other variables (e.g., light intensity), leading to possible spurious treatment effects (Hurlbert 1984). The conclusions of Hurlbert (1984) on pseudoreplication in growth chamber experiments have, however, been extensively criticized (e.g., Johnson et al. 2016; Oksanen 2001). Later, Hurlbert (2004) concluded that such experiments can be analyzed with standard statistical approaches if the interaction term is used as an estimate of the error term to test the main effect. Thus, in line with a range of other studies using similar settings for unreplicated gardens at different elevations (Gugger et al. 2015; Scheepens & Stocklin 2013) or growth chambers (Bezemer, Thompson & Jones 1998; Cavieres & Arroyo 2000; Matias & Jump 2014; Souther, Lechowicz & McGraw 2012; Zhang et al. 2014), we suggest that such studies are useful by allowing the separation of genetic differentiation of plants from their phenotypic plasticity and are also useful for assessing the importance of transgenerational plasticity in the system. For an extended discussion of this issue, see Text S4 in Münzbergová et al. (2017).

## 3 RESULTS

**Figure 1:**
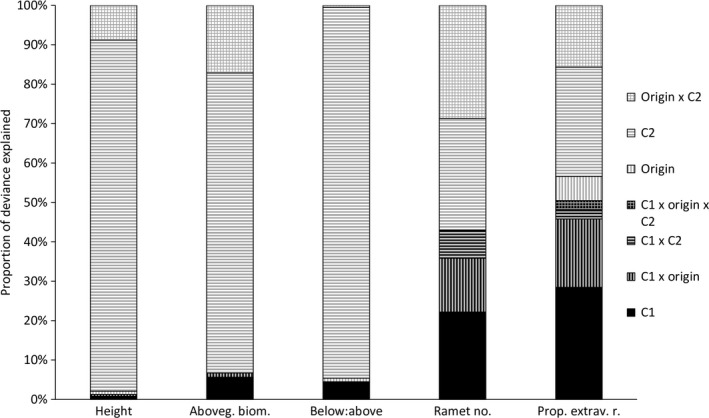
Importance of maternal conditions (effect of C1), original conditions and offspring conditions (C2), and their interactions for species performance. The values show proportion of the deviance explained by all significant variables in the model. Black parts of the columns indicate deviance explained by maternal conditions (C1) alone or in interaction with other factors; gray parts of the column indicate effect due to origin, offspring conditions (C2), or their interaction

Transgenerational effects (i.e., effects of C1 conditions and their interactions with other variables) accounted for more than 50% of the explained deviance in proportion of extravaginal ramets of the plants in C2 (Figure 1). Also, all the other variables in C2 were significantly affected by the transgenerational effects with the lowest transgenerational effects in above ground biomass, ratio between belowground and aboveground biomass and plant height (Figure 1). Most of the variation explained by the transgenerational effects was due to pure effects of conditions in C1 and the interaction of conditions in C1 and origin. In contrast, the interaction between conditions in C1 and C2 had only low explanatory power (Figure 1). However, the specific responses of plants were of different directions and intensities and did not confirm our expectation that plants exposed to certain conditions for half a year will subsequently perform better in those same conditions than in other conditions (Table 1, Table S1).

**Figure 2:**
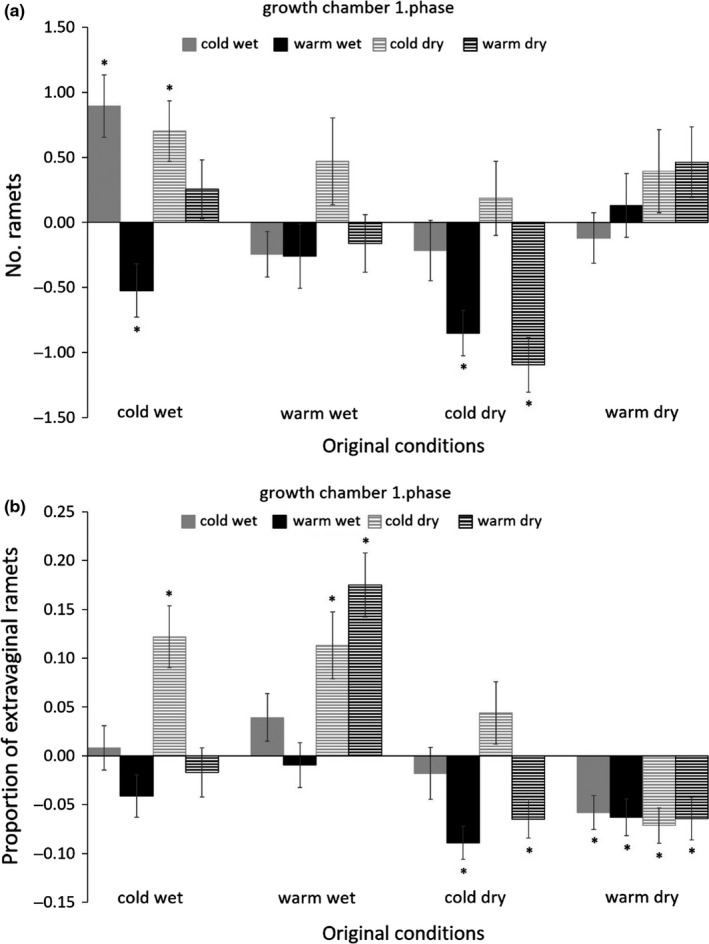
Effect of C1 conditions (1. phase) and conditions of origin on performance of the plants in C2. The values represent mean ± *SE* of deviation of each trait for individuals of each origin and each C1 cultivating conditions from mean value of the trait in the given C2 growth chamber. The effect of conditions in C2 is thus not shown, and the values represent deviations from the mean effect of the C2 growth chambers. Plant performance was measured as (a) ramet number and (b) proportion of extravaginal ramets. * indicates significant deviation from the mean trait value of the C2 growth chambers

Specifically, plants experiencing low temperature in C1 had significantly higher values of all the size measures in C2 (Table 1, Figure 2 and Fig. S1). In addition, plants experiencing drought in C1 had significantly more ramets and proportion of extravaginal ramets in C2 than plants experiencing high moisture in C1. Positive effect of high moisture in C1 on aboveground biomass in C2 was stronger in plants grown in cold conditions in C1 than in plants grown in warm C1 conditions. In addition, plants grew higher in C2 after experiencing moist and cold C1, while plants grew smaller in C2 after experiencing moist and warm C1 (Table 1, Figure 2 and Figs. S1, S2).

Plant performance in C2 was also affected by interaction of the conditions in C1 and original conditions in the field (Table 1). Specifically, plants originating from cold conditions had lower number of ramets and lower proportion of extravaginal ramets in C2 when exposed to warm conditions in C1, while plants originated from warm conditions did not show any significant response to C1 temperature. Plants that originated from wet conditions had lower proportion of extravaginal ramets in C2 when grown in wet C1, while plants from dry original conditions did not show any response to C1 moisture. In addition, plants from wet conditions increased their aboveground biomass in C2 with higher moisture in C1 much more than plants from dry conditions. Plants from warmer conditions were shorter in C2 than plants from colder conditions when grown in cold‐dry, warm‐dry, or warm‐wet conditions in C1, but not when grown in cold‐wet conditions in C1. Plants from warm conditions had fewer extravaginal ramets in C2 when experiencing cold‐dry C1 conditions than plants from cold conditions. In contrast, plants from warm conditions had more extravaginal ramets in C2 than plants from cold conditions if they experience cold‐wet C1 conditions. There was no significant effect of temperature of origin in plants experiencing warm C1 conditions on proportion of extravaginal ramets in C2. No other interactions between conditions in C1 and original conditions on plant performance in C2 were significant (Table 1, Figure 2 and Fig. S1). The effect of conditions in C1 also interacted with target conditions (C2) although these effects had always only low explanatory power (Figures 1, 2, Table 1, Table S1, Figs. S1 and S2). Plants experiencing the same conditions in C1 and C2 did not differ in their performance in C2 from the other plants in any of the traits (*p* > .05 in all cases).

**Figure 3:**
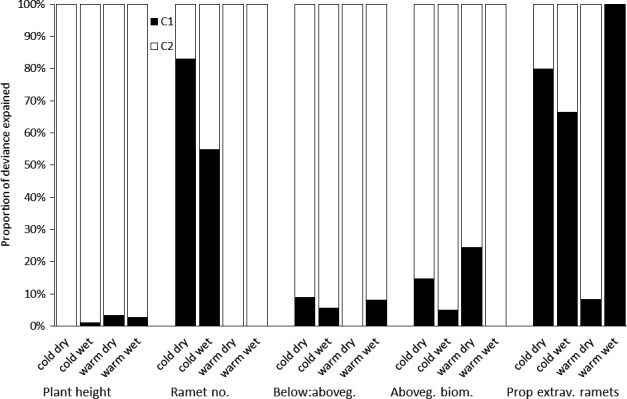
Effect of maternal conditions (C1) and conditions of cultivation (C2) on the different traits in each population separately. The values show proportion of the deviance explained by all significant variables in the model. Black bars of the columns indicate deviance explained by maternal conditions (C1) alone or in interaction with other factors; white bars indicate effect of offspring conditions (C2)

Comparison of the importance of transgenerational plasticity across populations demonstrated that transgenerational plasticity was least important in population from warm‐dry site. For ramet number, it was highly important in plant from cold sites but not in plant from warm sites (Figure 3).

## 4 DISCUSSION

The results of this study confirmed that transgenerational plasticity may be an important factor affecting species response to changing climate and may strongly interact with conditions of cultivation as well as with conditions of origin. The results also showed that such a transmission of effects is important also across clonal generations. For foraging‐related traits (proportion of extravaginal ramets), the variance explained by transgenerational effects and its interactions with target (C2) and origin were higher than the variance explained only by target (C2) and original conditions. This suggests that transgenerational effects may be the key factors driving performance of species of different origins in novel climates at least when it comes to resource acquisition by the plant.

Significant transgenerational effects detected in this study are in line with general expectation that such effects may be important for species performance in general (e.g., Guillaume et al., 2016; Herman & Sultan, 2011). The fact that we demonstrated these effects in clonal species adds to current growing body of the literature, suggesting that transgenerational plasticity may not only be important factor affecting performance of generatively reproducing species, but may be also important in clonal species (González, Dumalasová, Rosenthal, Skuhrovec, & Latzel, 2017; Gonzalez et al., 2016; Latzel & Klimesova, 2010).

The current result may also be linked to studies dealing with species acclimation ability. Acclimation is defined as facultative phenotypic response within juvenile or adult organisms that result in shift of reaction norms in response to environmental variation that occurs over a period of several days or longer. Phenotypic changes resulting from acclimation are reversible and repeatable in the lifetime of individuals (Beaman, White, & Seebacher, 2016). Note, however, that some authors use also the term transgenerational acclimation (e.g., Cahenzli & Erhardt, 2013; Donelson et al., 2012) to describe what we call transgenerational plasticity in this study. Recently, acclimation was suggested to be an important factor allowing animals to adapt to changing climatic conditions throughout their life (Beaman et al., 2016). For plants, it was shown that acclimation is a key factor allowing species to adjust their thermal optima for photosynthesis in several deciduous tree species (Gunderson, O'Hara, Campion, Walker, & Edwards, 2010). As we deal with clonal species, the patterns observed in our study may be in fact viewed, similar to the patterns in trees, also as acclimation within a single genetic individual.

In terms of species response to climate change, the results of this study suggest that short‐term exposure to novel climate will modify future species response to these conditions. However, the results did not confirm our expectation that plants exposed to certain conditions for half a year will subsequently perform better in those same conditions than in other conditions, that is, that transgenerational plasticity will be adaptive. This was evident from the result, showing that exposure to the same condition in the maternal and offspring phase did not affect plant performance in a different way than when the maternal and offspring conditions were different. This contrasts with a study showing that transgenerational plasticity may be adaptive in bryozoans in response to competition (Allen et al., 2008). Similarly, Herman and Sultan (2016) and González et al. (2017) showed that transgenerational plasticity may be adaptive in plant response to drought. González et al. (2017), however, also demonstrated that transgenerational plasticity is not adaptive in response to herbivory, suggesting that transgenerational plasticity may not always be adaptive.

The absence of indication of adaptive transgenerational plasticity may be also linked to the fact that the traits measured in our study were not proper measures of fitness. It was suggested that the various proxy traits measured on plants to describe their performance when assessing their adaptive potential may confuse the conclusions on adaptiveness of various processes in plant life (Marshall & Uller, 2007). Assessing life time fitness in our model is, however, almost impossible, given that it is long‐lived clonal species, life span of which may be up to several hundreds of years (Harberd, 1961; de Witte & Stocklin, 2010). The best proxy of plant fitness in this clonal species is probably the number of ramets, as it provides information on number of new clonal offspring. Even this trait, however, did not provide any indication of adaptiveness. González et al. (2017), however, found indication of adaptive transgenerational plasticity even when using data on plant size as a fitness measure. Even if the transgenerational plasticity is not adaptive, it still seems very important for affecting plant performance. The high proportion of variance explained by C1 conditions and their interaction with origin and C2 conditions in our study thus suggest that conclusions of studies exploring species response to novel climatic conditions over few weeks or months may be misleading, as they may not allow the plants to acclimate to the conditions in question.

Important outcome of our study is that for some traits the terms including the transgenerational effects either alone or in interaction with conditions of origin and conditions of cultivation accounted for more variation in the data than the terms accounting for species origin and for cultivating conditions alone. Such an effect was especially strong for proportion of extravaginal ramets. This trait has been previously shown to be strongly affected by environment of origin and showed strong interaction between environment of origin and cultivation in our previous study in the same system (Münzbergová et al. 2017). This trait is the least plastic traits and was most affected by the maternal environment. This result on stronger maternal (C1) effects in traits with lower plasticity contrasts with the conclusions of Hallsson, Chenoweth, and Bonduriansky (2012). They demonstrated in seed beetles that traits showing higher plasticity show stronger response to maternal environment than traits with stronger genetic determination. Hallsson et al. (2012) suggest that such a pattern can be expected for two different reasons. First, they suggest that whereas a nonplastic trait is subjected exclusively to genetic effects, a plastic trait can be influenced by the environment, including the developmental environment provided by parental phenotypes. Second, in highly plastic traits, additive genetic variance may be masked by high environmental variation and therefore difficult to detect (Hallsson et al., 2012). While both arguments seem reasonable, our study demonstrated that traits showing high plasticity show strong response to current environment and thus are not affected by maternal environment. The contrasting result may be explained by the fact that their traits were morphological traits likely initiated already during early stages of development in their model organisms, while our traits are purely growth related and may thus much more respond to the actual environment.

One of our hypotheses was that the transgenerational effects will depend on population origin. Indeed, we found many significant interactions between plant origin and conditions during the maternal environment. In contrast to Lampei et al. (2017), the interactions were very complex and thus hard to interpret. This could be explained by the fact that our study dealt with two interacting environmental gradients of origin, maternal as well as offspring generation. As the two gradients indeed strongly interacted with each other, this leads to very complex results in comparison with a single gradient studied in Lampei et al. (2017). Comparison of variance explained by transgenerational plasticity separately for each population and trait suggested that the lowest transgenerational effects were found in population from warm‐dry conditions, that is, in the population from the most favorable environment (c.f. Münzbergová et al. 2017).

A common procedure for experiments in multiple control conditions is to first cultivate the plants in standardized conditions for a certain period to remove the maternal effects (reviewed in Latzel, 2015). This treatment is often implemented in response to studies, demonstrating that maternal environment is an important driver of species performance (e.g., Galloway, 2001; Mousseau & Fox, 1998; Roach & Wulff, 1987; Wolf & Wade, 2009). This study demonstrated that the effect of such a precultivation may strongly interact with conditions of origin. By attempting to remove the maternal effects, we may thus strongly modify performance of the populations and do so to a different degree and direction in plants of different origins. Thus, the exact setting of the common cultivating conditions and duration of such a cultivation may strongly affect outcomes of subsequent experiments.

Understanding importance of transgenerational plasticity in response to climate is crucial as it may have significant effects on species ability to respond to novel climatic conditions likely to occur along with global climate change. It is expected that along with a continuous change in averages of climatic variables, the climate will simultaneously become more variable, extreme, and unpredictable (IPCC, 2014). In this study, we explored effects of transgenerational plasticity induced by cultivating the plants in different conditions for 6 months. This may simulate occurrence of a single extreme season and is thus in line with what is expected in terms of climate to occur. Our results are thus relevant for predicting species response to these fluctuating climatic conditions. The strong interactions between conditions during the maternal phase with conditions during the offspring phase and conditions of origin indicate that species performance under these novel conditions will be very hard to predict. This together with the lack of indication of adaptiveness of the transgenerational plasticity may suggest that transgenerational plasticity will be unlikely to improve species performance in novel climates.

In addition to responding to novel conditions in situ, species‐facing novel climates may also respond to climate change by migrating to different locations (e.g., Kokko & Lopez‐Sepulcre 2006; Nicotra et al. 2010). It can be expected that transgenerational plasticity will be important also under such a scenario. In this case, however, then transgenerational transmission should happen via seeds and not via clonal offspring as studied here. As generative reproduction is at least partly associated with resetting epigenetic memory (Verhoeven & Preite, 2014), an important mechanism responsible for transgenerational plasticity, the importance of transgenerational effects might be weaker in this situation.

## CONFLICT OF INTEREST

None declared.

## AUTHOR CONTRIBUTIONS

ZM and VH conceived the idea and designed methodology; VH and ZM collected the data; ZM analyzed the data; ZM wrote the manuscript. VH contributed critically to the drafts and gave final approval for publication.
