# Supplementary material for: Transgenerational plasticity as an important mechanism affecting response of clonal species to changing climate
Source: Ecol Evol. 2017 Jun 7;7(14):5236–47. doi: 10.1002/ece3.3105 (PMC5528211; doi:10.1002/ece3.3105)
Supplement: Supplementary file 1 [file ECE3-7-5236-s001.docx]

Supplementary file Table S1. Pair-wise correlation matrix between all the measured plant characteristics. The values represent correlation coefficients. Significant values (p ≤ 0.05) are in bold.

|  | Plant height | Ramet no. | Below:aboveg. | Aboveg. biom. | Belowg. biom. | Prop extrav. ramets | Rhizome biom. |
| --- | --- | --- | --- | --- | --- | --- | --- |
| Plant height | - | **0.153** | **-0.574** | **0.653** | **0.190** | **0.148** | **0.099** |
| Ramet no. | **0.153** | - | -0.007 | **0.553** | **0.579** | **0.285** | **0.327** |
| Below:aboveg. | **-0.574** | -0.007 | - | **-0.360** | **0.245** | -0.050 | 0.016 |
| Aboveg. biom. | **0.653** | **0.553** | **-0.360** | - | **0.701** | **0.158** | **0.158** |
| Belowg. biom. | **0.190** | **0.579** | **0.245** | **0.701** | - | **0.135** | **0.196** |
| Prop extrav. ramets | **0.148** | **0.285** | -0.050 | **0.158** | **0.135** | - | **0.736** |
| Rhizome biom. | **0.099** | **0.327** | 0.016 | **0.158** | **0.196** | **0.736** | - |
