# Supplementary material for: Transgenerational plasticity as an important mechanism affecting response of clonal species to changing climate
Source: Ecol Evol. 2017 Jun 7;7(14):5236–47. doi: 10.1002/ece3.3105 (PMC5528211; doi:10.1002/ece3.3105)
Supplement: Supplementary file 2 [file ECE3-7-5236-s002.docx]

Supplementary file Table S2. Triple interactions between temperature (T) and moisture (M) of origin (O), of the maternal (1) phase and of the offspring (2) phase of the experiment on all the measured species characteristics in the offspring phase (C2). Significant values (p ≤ 0.05) are in bold. Main effect and double interactions of the variables are shown in Table 1. Results marked with * are significant also after correcting for multiple testing. TO and MO thus represent the effects of origin, T1 and M1 represent the effects of C1 and T2 and M2 represent the effects of C2.

|  | Plant height | | Ramet no. | | Below:aboveg. | | Aboveg. biom. | | Prop extrav. ramets | |
| --- | --- | --- | --- | --- | --- | --- | --- | --- | --- | --- |
|  | F | p | F | p | F | p | F | p | Chi | p |
| TO x T1 x T2 | 0.03 | 0.857 | 0.32 | 0.574 | 0.17 | 0.678 | 0.10 | 0.758 | 0.57 | 0.543 |
| TO x T1 x MO | 0.39 | 0.535 | 1.11 | 0.293 | 0.29 | 0.070 | 0.01 | 0.930 | 0.14 | 0.712 |
| TO x T1 x M1 | **6.03** | **0.014** | 0.67 | 0.413 | 1.00 | 0.317 | 3.04 | 0.081 | **5.63** | **0.018** |
| TO x T1 x M2 | 0.13 | 0.714 | 0.37 | 0.544 | **4.25** | **0.039** | 1.94 | 0.163 | **3.93** | **0.047** |
| TO x MO x T2 | **18.53** | **<0.001*** | 0.56 | 0.456 | **4.64** | **0.031** | 0.43 | 0.510 | **8.31** | **0.004*** |
| TO x M1 x T2 | 1.66 | 0.198 | 0.57 | 0.450 | 2.16 | 0.141 | 0.72 | 0.400 | 2.21 | 0.137 |
| TO x M2 x T2 | **4.95** | **0.026** | <0.01 | 0.955 | 0.58 | 0.448 | 0.09 | 0.465 | 1.26 | 0.263 |
| TO x MO x M1 | 0.73 | 0.394 | 0.85 | 0.358 | **8.13** | **0.004*** | 0.55 | 0.458 | **4.93** | **0.026** |
| TO x MO x M2 | <0.01 | 0.992 | 0.02 | 0.880 | 0.70 | 0.403 | 0.07 | 0.799 | **9.08** | **0.003*** |
| TO x M1 x M2 | 0.05 | 0.827 | <0.01 | 0.987 | 0.04 | 0.851 | 0.11 | 0.737 | 0.53 | 0.456 |
| T1 x T2 x MO | 0.38 | 0.540 | <0.01 | 0.947 | 0.24 | 0.625 | 0.29 | 0.590 | 0.15 | 0.904 |
| T1 x T2 x M1 | 0.58 | 0.446 | 0.03 | 0.866 | 0.17 | 0.679 | 0.01 | 0.933 | 0.40 | 0.527 |
| T1 x T2 x M2 | 0.08 | 0.776 | 1.77 | 0.183 | 0.04 | 0.846 | 2.67 | 0.102 | **4.23** | **0.040** |
| T1 x MO x M1 | 0.02 | 0.886 | 2.81 | 0.093 | 0.22 | 0.637 | 0.13 | 0.723 | 0.01 | 0.932 |
| T1 x M1 x M2 | 0.35 | 0.556 | **4.93** | **0.026** | 0.09 | 0.767 | 1.30 | 0.255 | 3.24 | 0.071 |
| T2 x MO x M1 | 2.44 | 0.118 | 1.02 | 0.312 | 0.08 | 0.783 | 0.09 | 0.768 | 0.02 | 0.899 |
| T2 x MO x M2 | **33.52** | **<0.001*** | 1.07 | 0.301 | 0.32 | 0.572 | **11.49** | **<0.001*** | 0.03 | 0.865 |
| T2 x M1 x M2 | 1.29 | 0.256 | 0.23 | 0.630 | 0.60 | 0.439 | 0.13 | 0.716 | 1.02 | 0.313 |
| MO x M1 x M2 | 0.04 | 0.890 | 0.09 | 0.750 | 0.11 | 0.680 | 0.50 | 0.840 | 0.04 | 0.891 |
