# Supplementary material for: Transgenerational plasticity as an important mechanism affecting response of clonal species to changing climate
Source: Ecol Evol. 2017 Jun 7;7(14):5236–47. doi: 10.1002/ece3.3105 (PMC5528211; doi:10.1002/ece3.3105)

Supplementary file Figure S3. The effect of climate of origin (shown on the X-axes), climate in C1 (growth chamber 1. phase, represented by the different lines) and climate in which the plant is currently cultivated, i.e. C2 (growth chamber 2. phase, represented by the different panels) on A) plant height, B) ramet number, C) aboveground biomass, D) proportion of aboveground and belowground biomass and E) proportion of extravaginal ramets.

A)


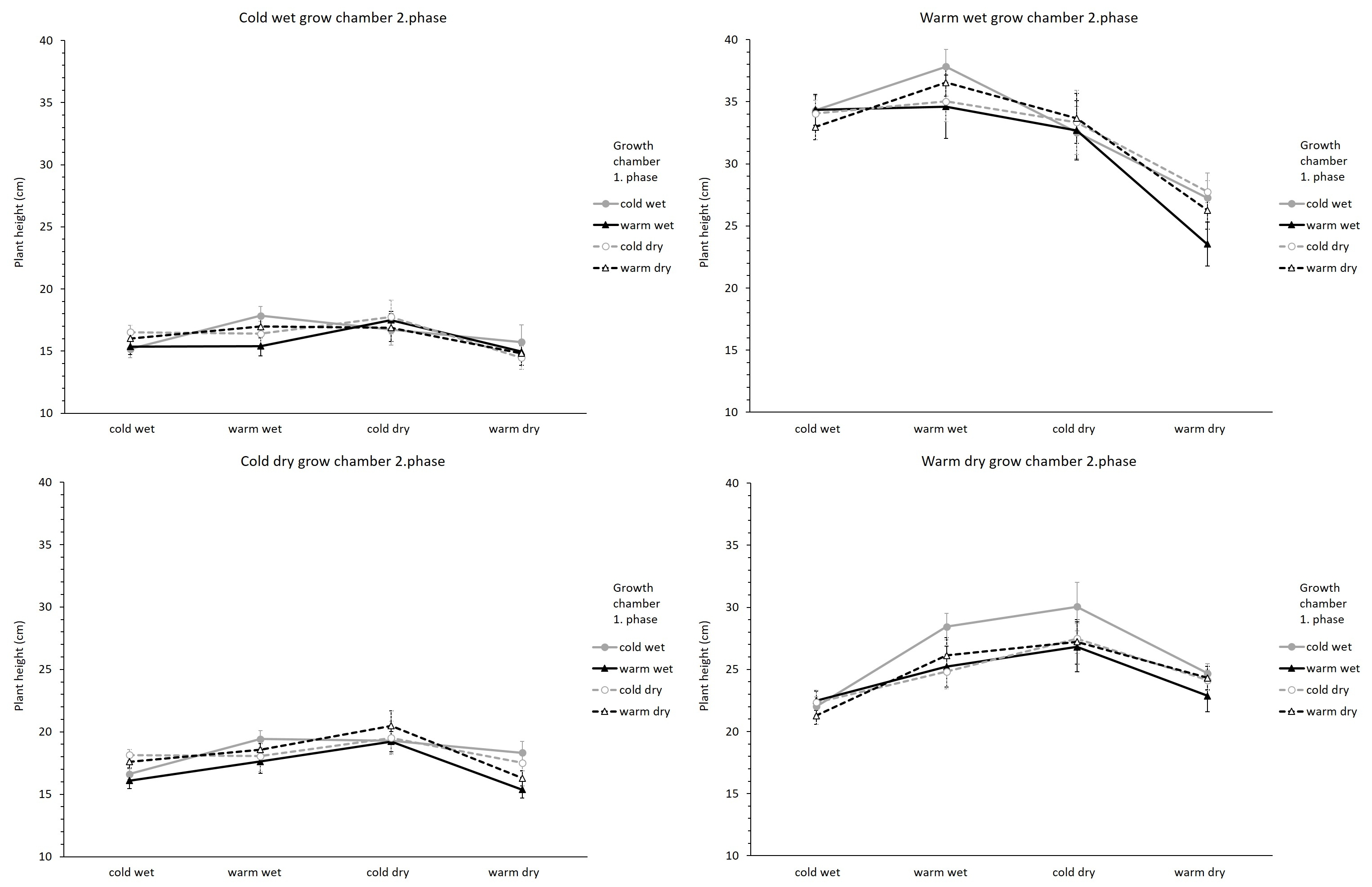


B)


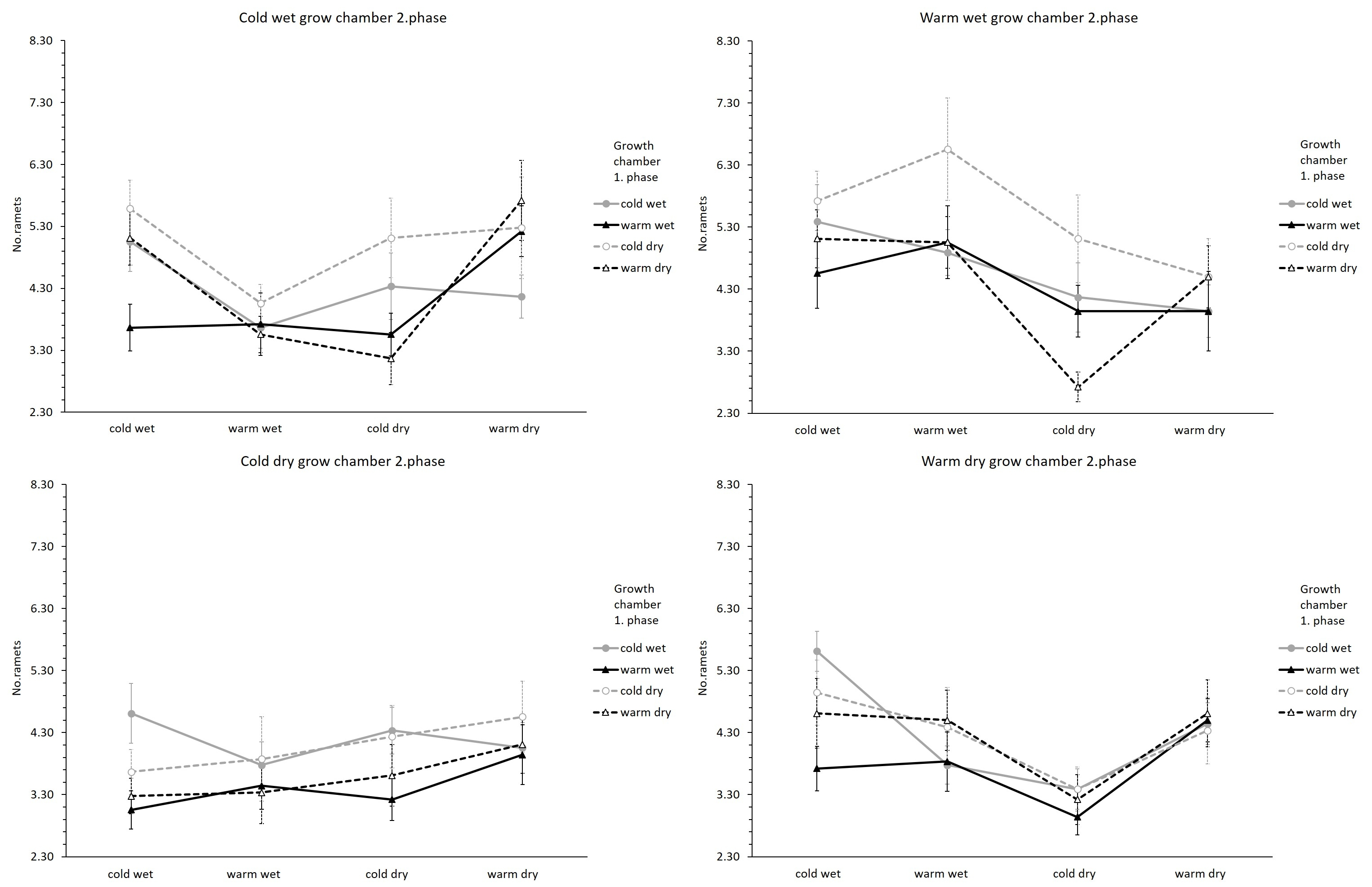


C)


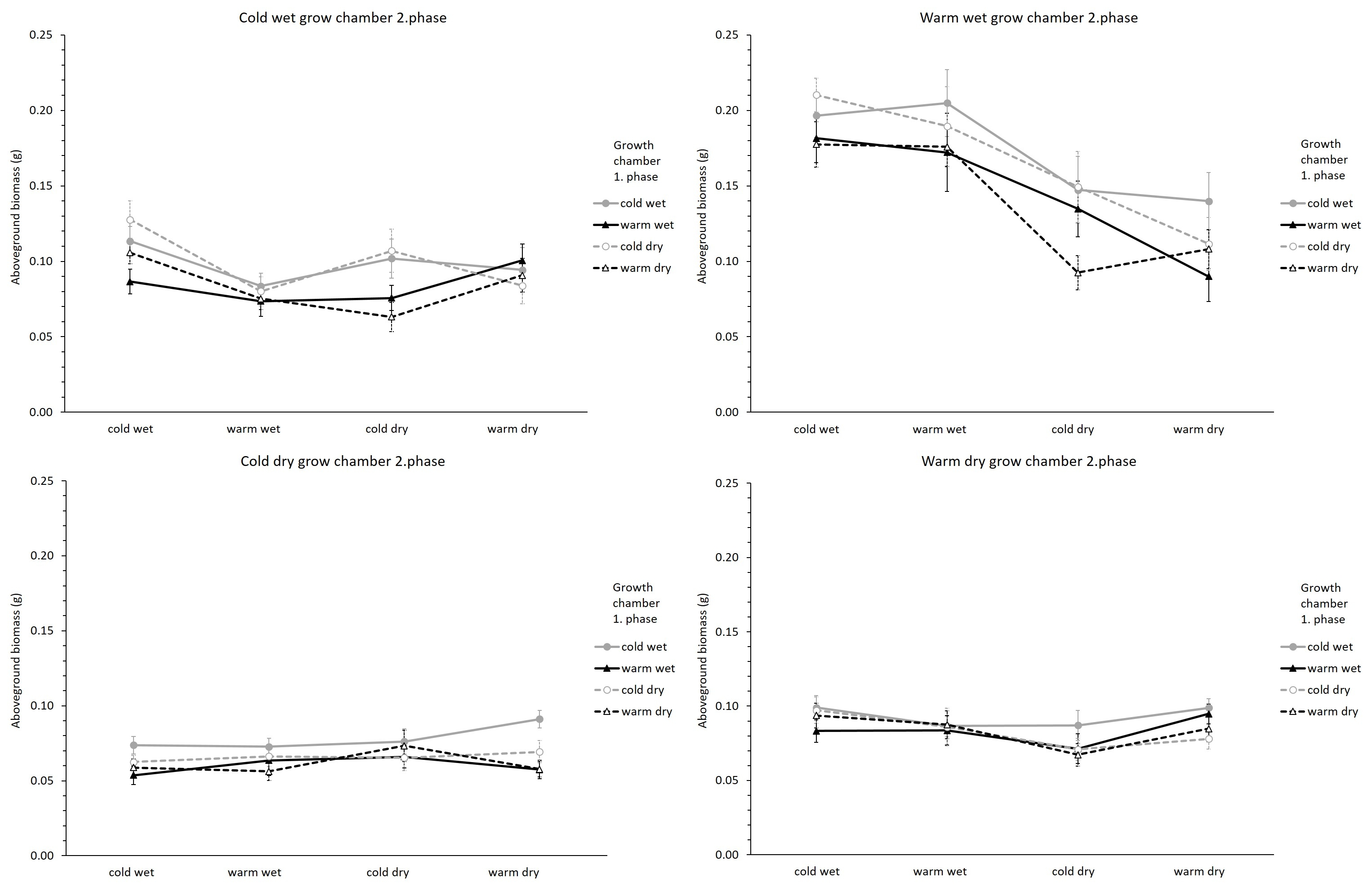


D)


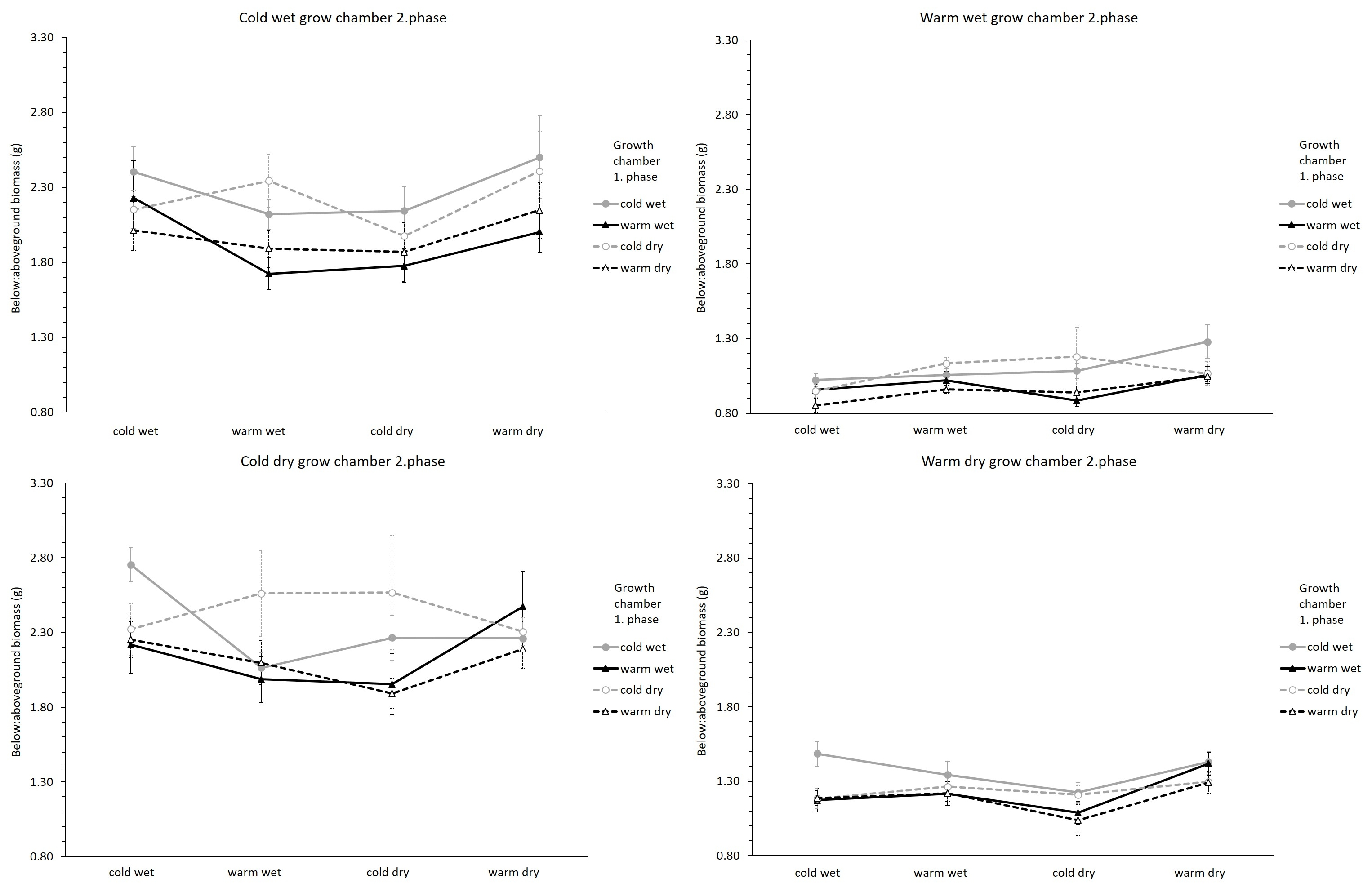


E)


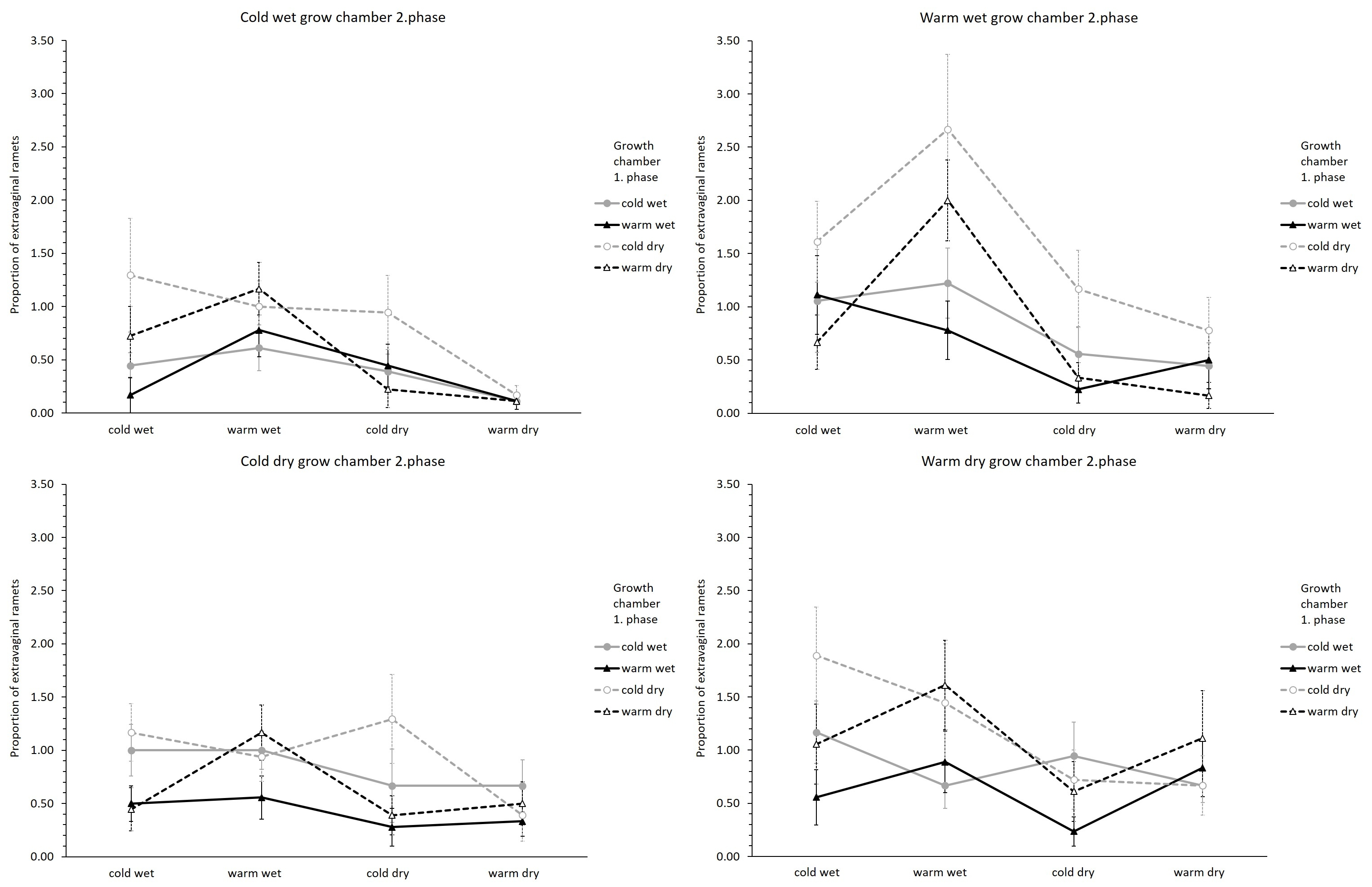

Supplement: Supplementary file 3 [file ECE3-7-5236-s003.docx]
