# Supplementary material for: Transgenerational plasticity as an important mechanism affecting response of clonal species to changing climate
Source: Ecol Evol. 2017 Jun 7;7(14):5236–47. doi: 10.1002/ece3.3105 (PMC5528211; doi:10.1002/ece3.3105)

Supplementary file Figure S4. The effect of conditions in C1 and conditions of origin on performance of the plants in C2. The values represent mean±SE of deviation of each trait for individuals of each origin and each C1 cultivating conditions from mean value of the trait in the given C2 growth chamber. The effect of conditions in C2 is thus not shown and the values represent deviations from the mean C2 effects. Plant performance was measured as A) plant height, B) aboveground biomass and C) proportion of aboveground and belowground biomass. * indicates significant deviation from the mean trait value in the given C2 growth chamber.

A)


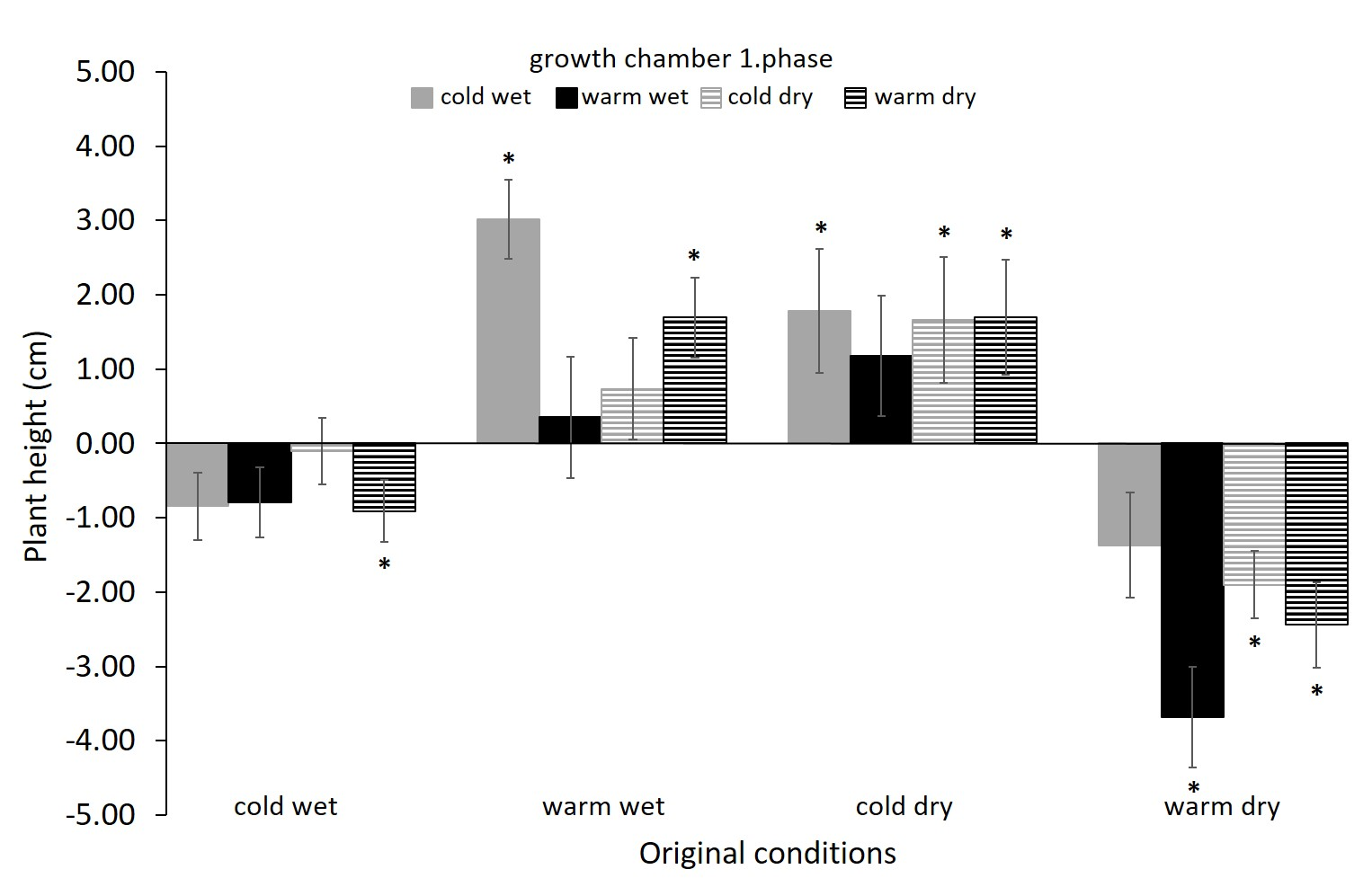


B)


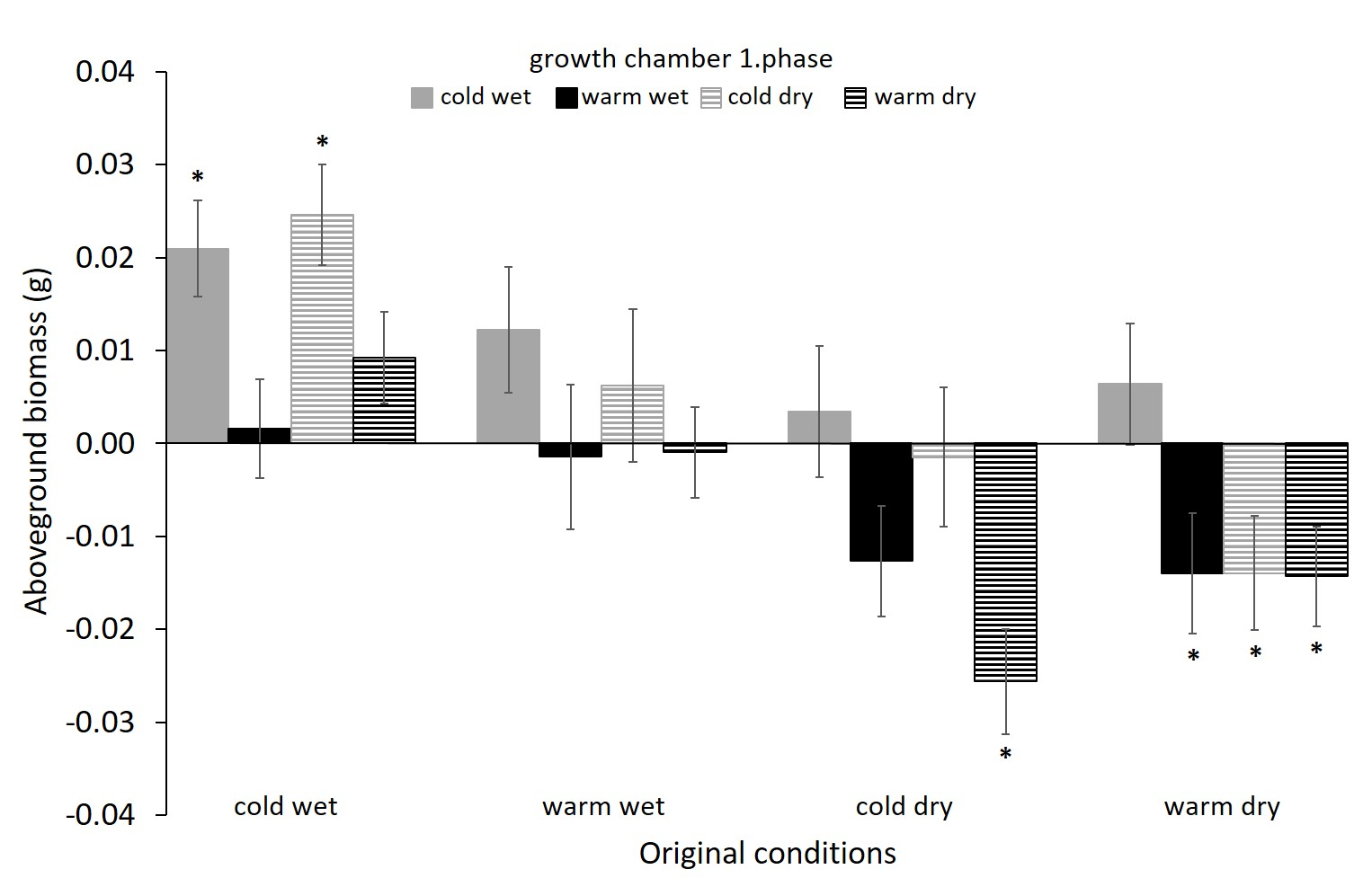


C)


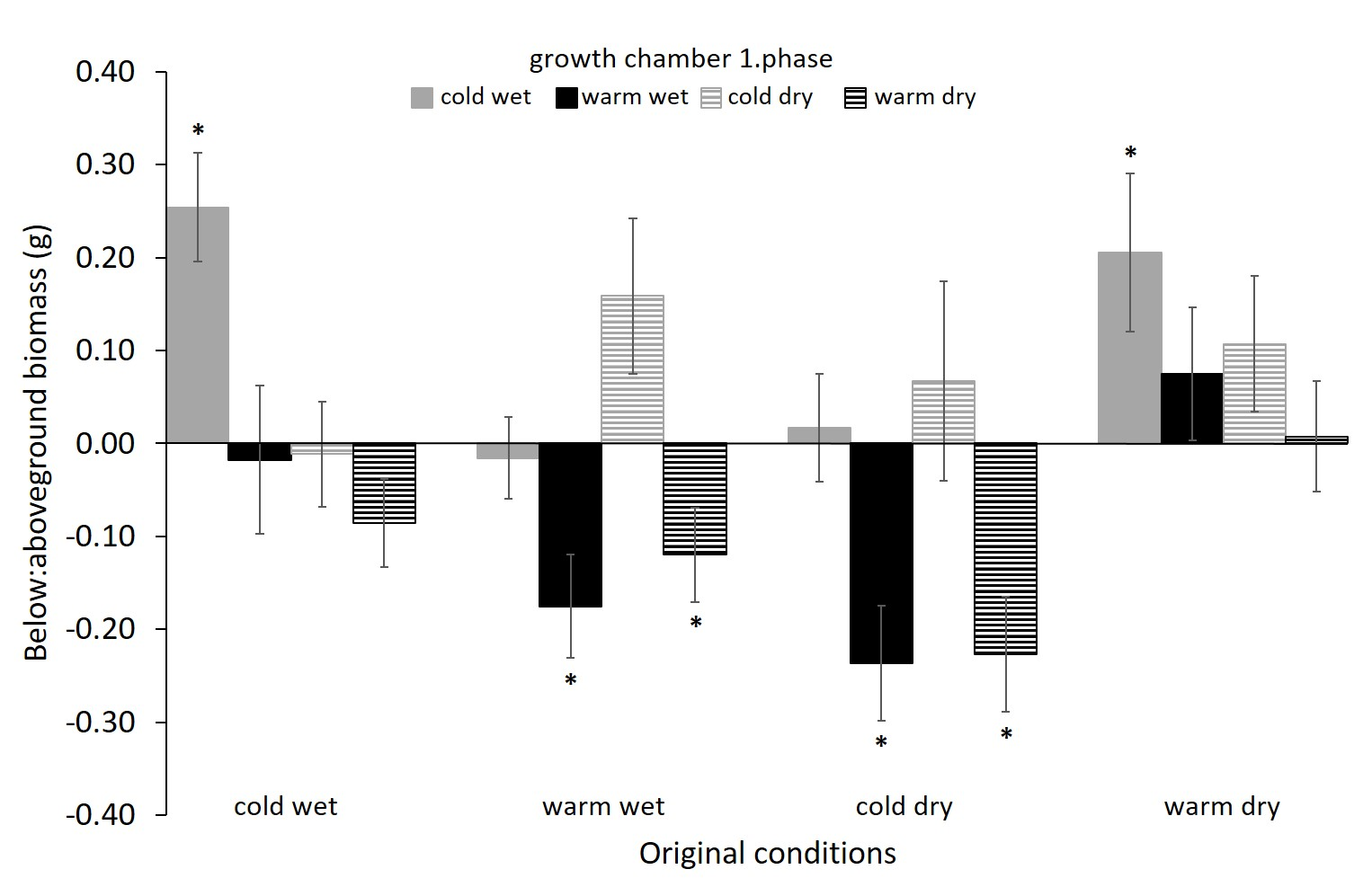

Supplement: Supplementary file 4 [file ECE3-7-5236-s004.docx]
